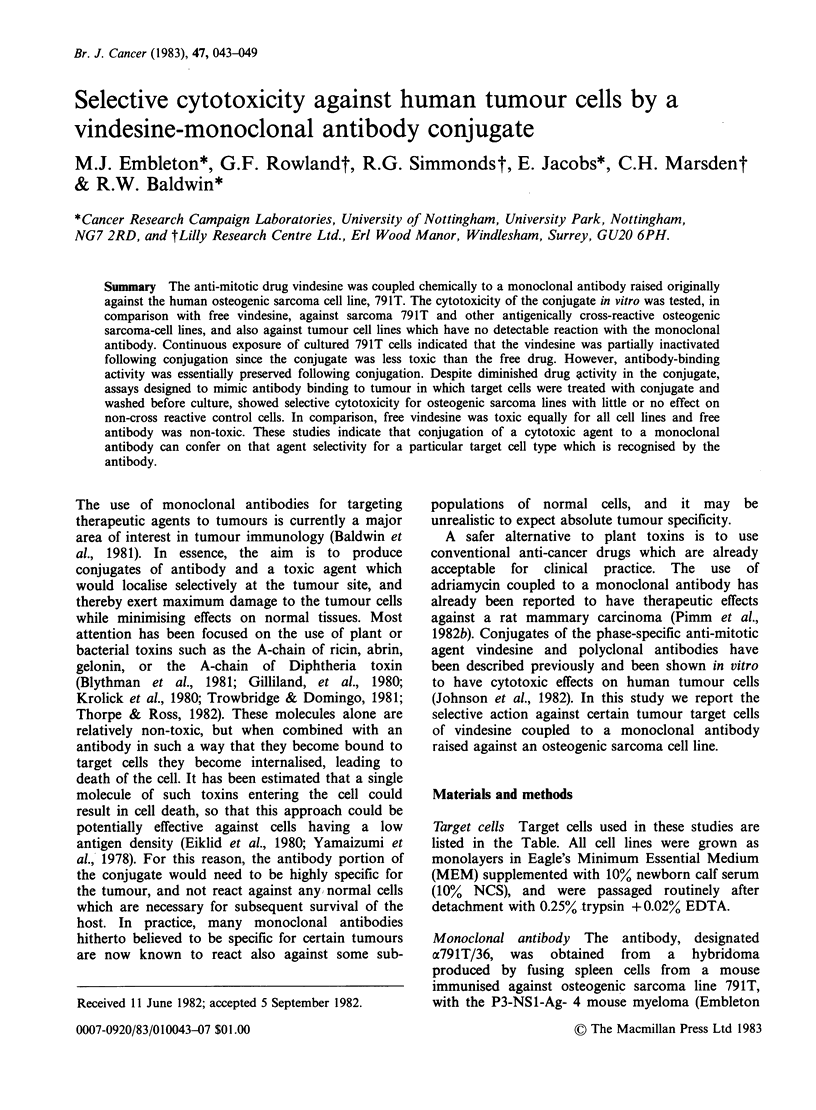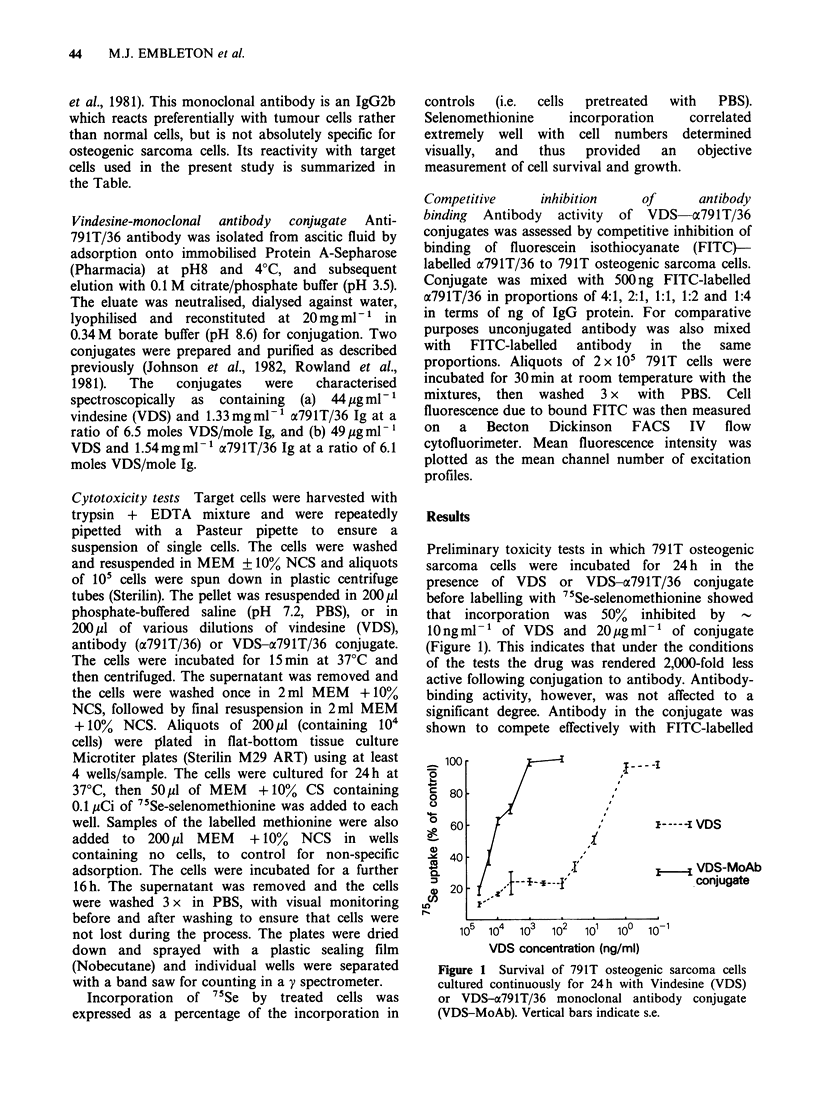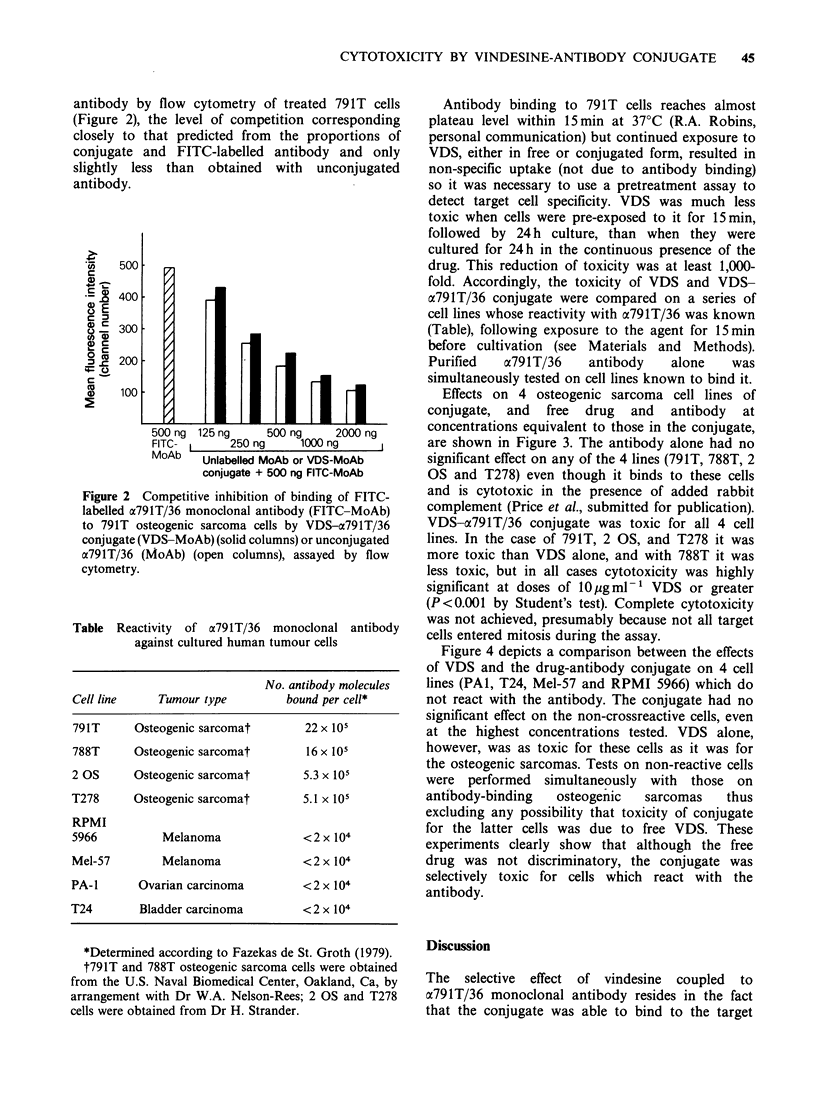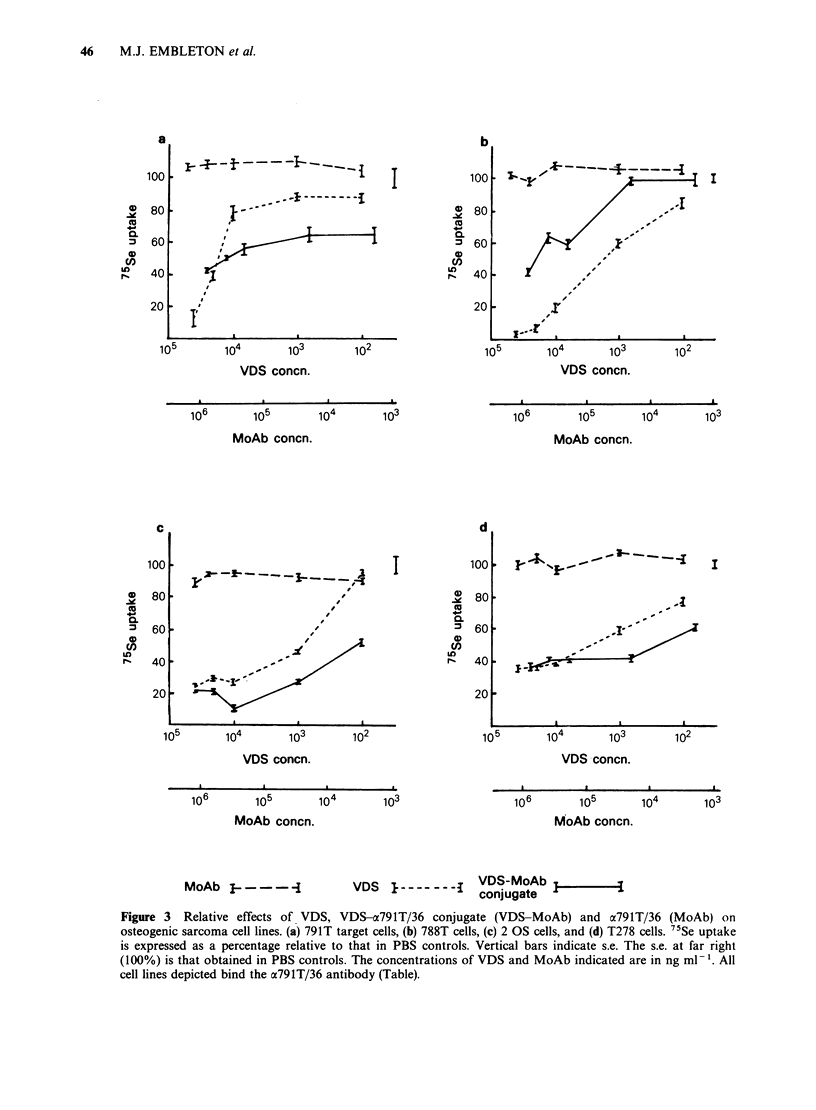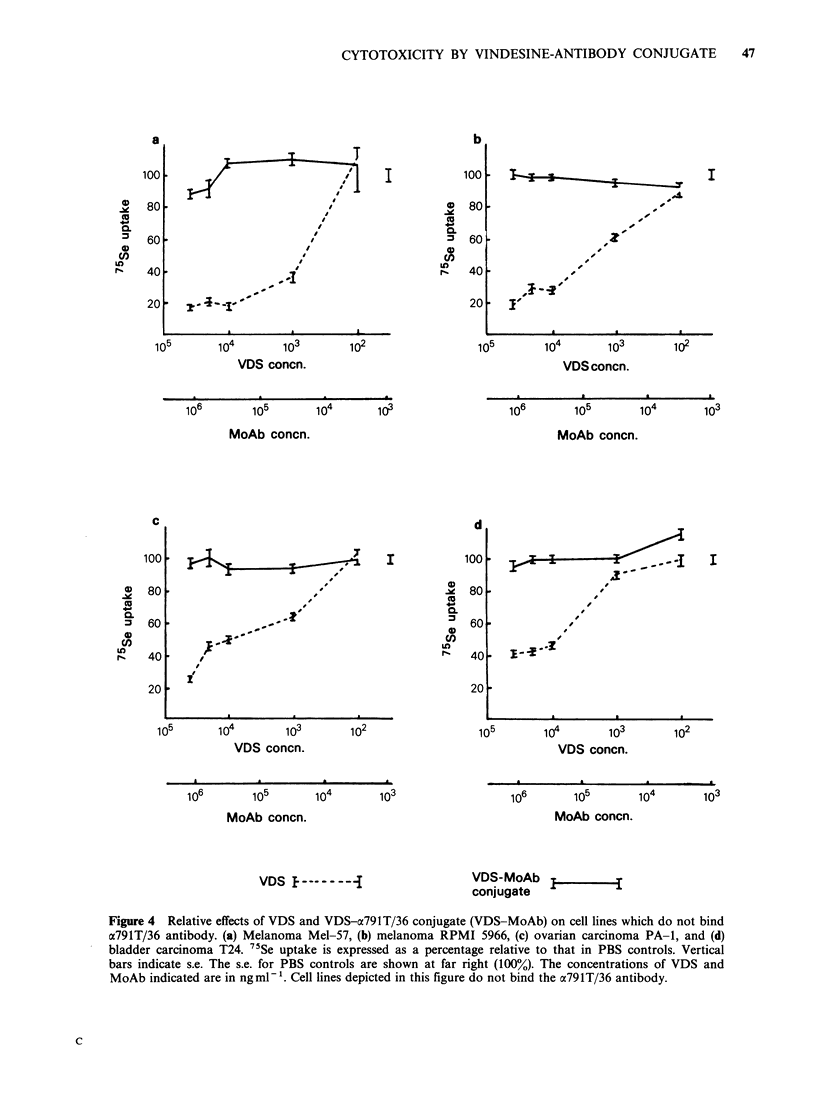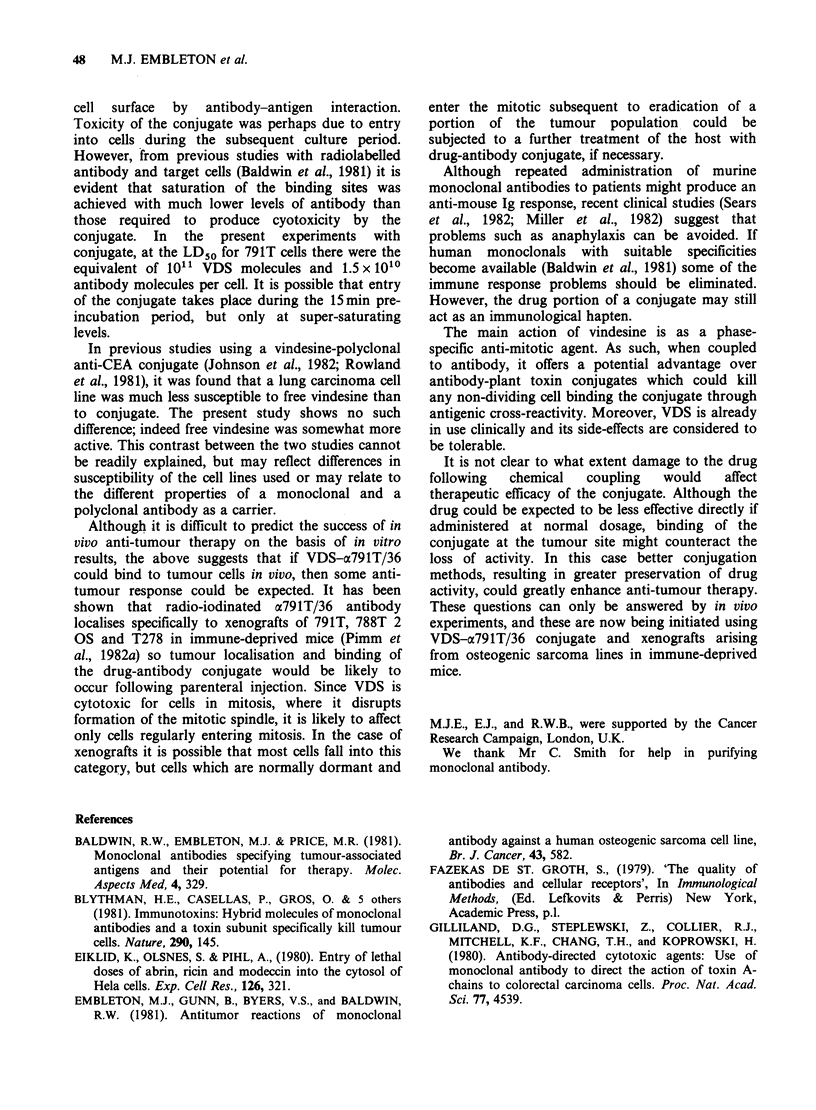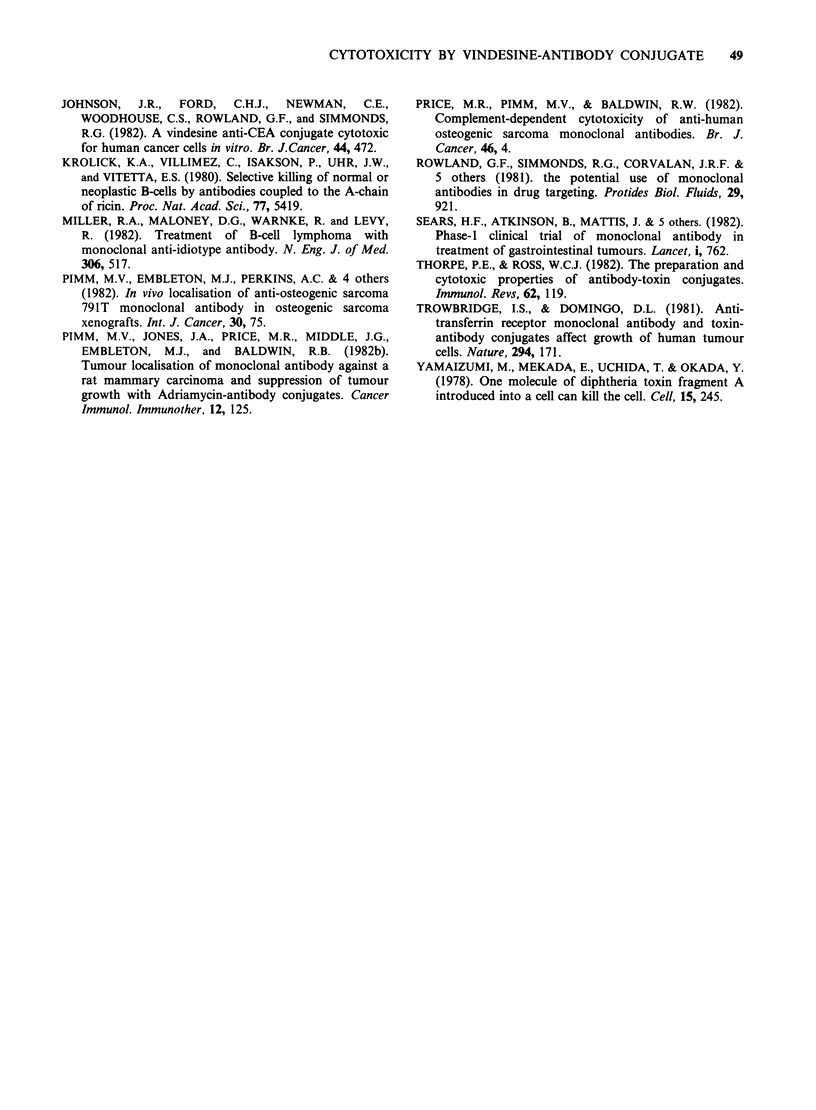# Selective cytotoxicity against human tumour cells by a vindesine-monoclonal antibody conjugate.

**DOI:** 10.1038/bjc.1983.5

**Published:** 1983-01

**Authors:** M. J. Embleton, G. F. Rowland, R. G. Simmonds, E. Jacobs, C. H. Marsden, R. W. Baldwin

## Abstract

The anti-mitotic drug vindesine was coupled chemically to a monoclonal antibody raised originally against the human osteogenic sarcoma cell line, 791T. The cytotoxicity of the conjugate in vitro was tested, in comparison with free vindesine, against sarcoma 791T and other antigenically cross-reactive osteogenic sarcoma-cell lines, and also against tumour cell lines which have no detectable reaction with the monoclonal antibody. Continuous exposure of cultured 791T cells indicated that the vindesine was partially inactivated following conjugation since the conjugate was less toxic than the free drug. However, antibody-binding activity was essentially preserved following conjugation. Despite diminished drug activity in the conjugate, assays designed to mimic antibody binding to tumour in which target cells were treated with conjugate and washed before culture, showed selective cytotoxicity for osteogenic sarcoma lines with little or no effect on non-cross reactive control cells. In comparison, free vindesine was toxic equally for all cell lines and free antibody was non-toxic. These studies indicate that conjugation of a cytotoxic agent to a monoclonal antibody can confer on that agent selectivity for a particular target cell type which is recognised by the antibody.


*Br. J. Cancer* (1983), **47**, 043–049

### Selective cytotoxicity against human tumour cells by a vindesine-monoclonal antibody conjugate

M.J. Embleton*, G.F. Rowland†, R.G. Simmonds†, E. Jacobs*, C.H. Marsden† & R.W. Baldwin*

**Cancer Research Campaign Laboratories, University of Nottingham, University Park, Nottingham, NG7 2RD, and †Lilly Research Centre Ltd., Erl Wood Manor, Windlesham, Surrey, GU20 6PH.*

**Summary** The anti-mitotic drug vindesine was coupled chemically to a monoclonal antibody raised originally against the human osteogenic sarcoma cell line, 791T. The cytotoxicity of the conjugate *in vitro* was tested, in comparison with free vindesine, against sarcoma 791T and other antigenically cross-reactive osteogenic sarcoma-cell lines, and also against tumour cell lines which have no detectable reaction with the monoclonal antibody. Continuous exposure of cultured 791T cells indicated that the vindesine was partially inactivated following conjugation since the conjugate was less toxic than the free drug. However, antibody-binding activity was essentially preserved following conjugation. Despite diminished drug activity in the conjugate, assays designed to mimic antibody binding to tumour in which target cells were treated with conjugate and washed before culture, showed selective cytotoxicity for osteogenic sarcoma lines with little or no effect on non-cross reactive control cells. In comparison, free vindesine was toxic equally for all cell lines and free antibody was non-toxic. These studies indicate that conjugation of a cytotoxic agent to a monoclonal antibody can confer on that agent selectivity for a particular target cell type which is recognised by the antibody.

The use of monoclonal antibodies for targeting therapeutic agents to tumours is currently a major area of interest in tumour immunology (Baldwin *et al.*, 1981). In essence, the aim is to produce conjugates of antibody and a toxic agent which would localise selectively at the tumour site, and thereby exert maximum damage to the tumour cells while minimising effects on normal tissues. Most attention has been focused on the use of plant or bacterial toxins such as the A-chain of ricin, abrin, gelonin, or the A-chain of Diphtheria toxin (Blythman *et al.*, 1981; Gilliland, *et al.*, 1980; Krolick *et al.*, 1980; Trowbridge & Domingo, 1981; Thorpe & Ross, 1982). These molecules alone are relatively non-toxic, but when combined with an antibody in such a way that they become bound to target cells they become internalised, leading to death of the cell. It has been estimated that a single molecule of such toxins entering the cell could result in cell death, so that this approach could be potentially effective against cells having a low antigen density (Eiklid *et al.*, 1980; Yamaizumi *et al.*, 1978). For this reason, the antibody portion of the conjugate would need to be highly specific for the tumour, and not react against any normal cells which are necessary for subsequent survival of the host. In practice, many monoclonal antibodies hitherto believed to be specific for certain tumours are now known to react also against some sub-populations of normal cells, and it may be unrealistic to expect absolute tumour specificity.

A safer alternative to plant toxins is to use conventional anti-cancer drugs which are already acceptable for clinical practice. The use of adriamycin coupled to a monoclonal antibody has already been reported to have therapeutic effects against a rat mammary carcinoma (Pimm *et al.*, 1982*b*). Conjugates of the phase-specific anti-mitotic agent vindesine and polyclonal antibodies have been described previously and been shown *in vitro* to have cytotoxic effects on human tumour cells (Johnson *et al.*, 1982). In this study we report the selective action against certain tumour target cells of vindesine coupled to a monoclonal antibody raised against an osteogenic sarcoma cell line.

### Materials and methods

*Target cells* Target cells used in these studies are listed in the Table. All cell lines were grown as monolayers in Eagle's Minimum Essential Medium (MEM) supplemented with 10% newborn calf serum (10% NCS), and were passaged routinely after detachment with 0.25% trypsin +0.02% EDTA.

*Monoclonal antibody* The antibody, designated α791T/36, was obtained from a hybridoma produced by fusing spleen cells from a mouse immunised against osteogenic sarcoma line 791T, with the P3-NS1-Ag- 4 mouse myeloma (Embleton

Received 11 June 1982; accepted 5 September 1982.

0007-0920/83/010043–07 $01.00

© The Macmillan Press Ltd 1983

*et al.*, 1981). This monoclonal antibody is an IgG2b which reacts preferentially with tumour cells rather than normal cells, but is not absolutely specific for osteogenic sarcoma cells. Its reactivity with target cells used in the present study is summarized in the Table.

*Vindesine-monoclonal antibody conjugate* Anti-791T/36 antibody was isolated from ascitic fluid by adsorption onto immobilised Protein A-Sepharose (Pharmacia) at pH8 and 4°C, and subsequent elution with 0.1 M citrate/phosphate buffer (pH 3.5). The eluate was neutralised, dialysed against water, lyophilised and reconstituted at $20\,mg\,ml^{-1}$ in 0.34 M borate buffer (pH 8.6) for conjugation. Two conjugates were prepared and purified as described previously (Johnson *et al.*, 1982, Rowland *et al.*, 1981). The conjugates were characterised spectroscopically as containing (a) $44\,\mu g\,ml^{-1}$ vindesine (VDS) and $1.33\,mg\,ml^{-1}$ $\alpha$791T/36 Ig at a ratio of 6.5 moles VDS/mole Ig, and (b) $49\,\mu g\,ml^{-1}$ VDS and $1.54\,mg\,ml^{-1}$ $\alpha$791T/36 Ig at a ratio of 6.1 moles VDS/mole Ig.

*Cytotoxicity tests* Target cells were harvested with trypsin + EDTA mixture and were repeatedly pipetted with a Pasteur pipette to ensure a suspension of single cells. The cells were washed and resuspended in MEM $\pm 10\%$ NCS and aliquots of $10^5$ cells were spun down in plastic centrifuge tubes (Sterilin). The pellet was resuspended in $200\,\mu l$ phosphate-buffered saline (pH 7.2, PBS), or in $200\,\mu l$ of various dilutions of vindesine (VDS), antibody ($\alpha$791T/36) or VDS-$\alpha$791T/36 conjugate. The cells were incubated for 15 min at 37°C and then centrifuged. The supernatant was removed and the cells were washed once in 2 ml MEM $+10\%$ NCS, followed by final resuspension in 2 ml MEM $+10\%$ NCS. Aliquots of $200\,\mu l$ (containing $10^4$ cells) were plated in flat-bottom tissue culture Microtiter plates (Sterilin M29 ART) using at least 4 wells/sample. The cells were cultured for 24 h at 37°C, then $50\,\mu l$ of MEM $+10\%$ CS containing $0.1\,\mu Ci$ of $^{75}Se$-selenomethionine was added to each well. Samples of the labelled methionine were also added to $200\,\mu l$ MEM $+10\%$ NCS in wells containing no cells, to control for non-specific adsorption. The cells were incubated for a further 16 h. The supernatant was removed and the cells were washed $3\times$ in PBS, with visual monitoring before and after washing to ensure that cells were not lost during the process. The plates were dried down and sprayed with a plastic sealing film (Nobecutane) and individual wells were separated with a band saw for counting in a $\gamma$ spectrometer.

Incorporation of $^{75}Se$ by treated cells was expressed as a percentage of the incorporation in controls (i.e. cells pretreated with PBS). Selenomethionine incorporation correlated extremely well with cell numbers determined visually, and thus provided an objective measurement of cell survival and growth.

*Competitive inhibition of antibody binding* Antibody activity of VDS—$\alpha$791T/36 conjugates was assessed by competitive inhibition of binding of fluorescein isothiocyanate (FITC)—labelled $\alpha$791T/36 to 791T osteogenic sarcoma cells. Conjugate was mixed with 500 ng FITC-labelled $\alpha$791T/36 in proportions of 4:1, 2:1, 1:1, 1:2 and 1:4 in terms of ng of IgG protein. For comparative purposes unconjugated antibody was also mixed with FITC-labelled antibody in the same proportions. Aliquots of $2\times10^5$ 791T cells were incubated for 30 min at room temperature with the mixtures, then washed $3\times$ with PBS. Cell fluorescence due to bound FITC was then measured on a Becton Dickinson FACS IV flow cytofluorimeter. Mean fluorescence intensity was plotted as the mean channel number of excitation profiles.

### Results

Preliminary toxicity tests in which 791T osteogenic sarcoma cells were incubated for 24 h in the presence of VDS or VDS-$\alpha$791T/36 conjugate before labelling with $^{75}Se$-selenomethionine showed that incorporation was 50% inhibited by $\sim 10\,ng\,ml^{-1}$ of VDS and $20\,\mu g\,ml^{-1}$ of conjugate (Figure 1). This indicates that under the conditions of the tests the drug was rendered 2,000-fold less active following conjugation to antibody. Antibody-binding activity, however, was not affected to a significant degree. Antibody in the conjugate was shown to compete effectively with FITC-labelled

**Figure 1** Survival of 791T osteogenic sarcoma cells cultured continuously for 24 h with Vindesine (VDS) or VDS-$\alpha$791T/36 monoclonal antibody conjugate (VDS-MoAb). Vertical bars indicate s.e.

antibody by flow cytometry of treated 791T cells (Figure 2), the level of competition corresponding closely to that predicted from the proportions of conjugate and FITC-labelled antibody and only slightly less than obtained with unconjugated antibody.

**Figure 2** Competitive inhibition of binding of FITC-labelled α791T/36 monoclonal antibody (FITC–MoAb) to 791T osteogenic sarcoma cells by VDS–α791T/36 conjugate (VDS–MoAb) (solid columns) or unconjugated α791T/36 (MoAb) (open columns), assayed by flow cytometry.

**Table** Reactivity of α791T/36 monoclonal antibody against cultured human tumour cells

| *Cell line* | *Tumour type* | *No. antibody molecules bound per cell** |
|---|---|---|
| 791T | Osteogenic sarcoma† | $22 \times 10^5$ |
| 788T | Osteogenic sarcoma† | $16 \times 10^5$ |
| 2 OS | Osteogenic sarcoma† | $5.3 \times 10^5$ |
| T278 | Osteogenic sarcoma† | $5.1 \times 10^5$ |
| RPMI 5966 | Melanoma | $<2 \times 10^4$ |
| Mel-57 | Melanoma | $<2 \times 10^4$ |
| PA-1 | Ovarian carcinoma | $<2 \times 10^4$ |
| T24 | Bladder carcinoma | $<2 \times 10^4$ |

*Determined according to Fazekas de St. Groth (1979).
†791T and 788T osteogenic sarcoma cells were obtained from the U.S. Naval Biomedical Center, Oakland, Ca, by arrangement with Dr W.A. Nelson-Rees; 2 OS and T278 cells were obtained from Dr H. Strander.

Antibody binding to 791T cells reaches almost plateau level within 15 min at 37°C (R.A. Robins, personal communication) but continued exposure to VDS, either in free or conjugated form, resulted in non-specific uptake (not due to antibody binding) so it was necessary to use a pretreatment assay to detect target cell specificity. VDS was much less toxic when cells were pre-exposed to it for 15 min, followed by 24 h culture, than when they were cultured for 24 h in the continuous presence of the drug. This reduction of toxicity was at least 1,000-fold. Accordingly, the toxicity of VDS and VDS–α791T/36 conjugate were compared on a series of cell lines whose reactivity with α791T/36 was known (Table), following exposure to the agent for 15 min before cultivation (see Materials and Methods). Purified α791T/36 antibody alone was simultaneously tested on cell lines known to bind it.

Effects on 4 osteogenic sarcoma cell lines of conjugate, and free drug and antibody at concentrations equivalent to those in the conjugate, are shown in Figure 3. The antibody alone had no significant effect on any of the 4 lines (791T, 788T, 2 OS and T278) even though it binds to these cells and is cytotoxic in the presence of added rabbit complement (Price *et al.*, submitted for publication). VDS–α791T/36 conjugate was toxic for all 4 cell lines. In the case of 791T, 2 OS, and T278 it was more toxic than VDS alone, and with 788T it was less toxic, but in all cases cytotoxicity was highly significant at doses of $10\,\mu g\,ml^{-1}$ VDS or greater ($P<0.001$ by Student's test). Complete cytotoxicity was not achieved, presumably because not all target cells entered mitosis during the assay.

Figure 4 depicts a comparison between the effects of VDS and the drug-antibody conjugate on 4 cell lines (PA1, T24, Mel-57 and RPMI 5966) which do not react with the antibody. The conjugate had no significant effect on the non-crossreactive cells, even at the highest concentrations tested. VDS alone, however, was as toxic for these cells as it was for the osteogenic sarcomas. Tests on non-reactive cells were performed simultaneously with those on antibody-binding osteogenic sarcomas thus excluding any possibility that toxicity of conjugate for the latter cells was due to free VDS. These experiments clearly show that although the free drug was not discriminatory, the conjugate was selectively toxic for cells which react with the antibody.

### Discussion

The selective effect of vindesine coupled to α791T/36 monoclonal antibody resides in the fact that the conjugate was able to bind to the target

**Figure 3** Relative effects of VDS, VDS–α791T/36 conjugate (VDS–MoAb) and α791T/36 (MoAb) on osteogenic sarcoma cell lines. (**a**) 791T target cells, (**b**) 788T cells, (**c**) 2 OS cells, and (**d**) T278 cells. $^{75}$Se uptake is expressed as a percentage relative to that in PBS controls. Vertical bars indicate s.e. The s.e. at far right (100%) is that obtained in PBS controls. The concentrations of VDS and MoAb indicated are in ng ml$^{-1}$. All cell lines depicted bind the α791T/36 antibody (Table).

**Figure 4** Relative effects of VDS and VDS–α791T/36 conjugate (VDS–MoAb) on cell lines which do not bind α791T/36 antibody. (**a**) Melanoma Mel–57, (**b**) melanoma RPMI 5966, (**c**) ovarian carcinoma PA–1, and (**d**) bladder carcinoma T24. $^{75}$Se uptake is expressed as a percentage relative to that in PBS controls. Vertical bars indicate s.e. The s.e. for PBS controls are shown at far right (100%). The concentrations of VDS and MoAb indicated are in ng ml$^{-1}$. Cell lines depicted in this figure do not bind the α791T/36 antibody.

cell surface by antibody–antigen interaction. Toxicity of the conjugate was perhaps due to entry into cells during the subsequent culture period. However, from previous studies with radiolabelled antibody and target cells (Baldwin *et al.*, 1981) it is evident that saturation of the binding sites was achieved with much lower levels of antibody than those required to produce cyotoxicity by the conjugate. In the present experiments with conjugate, at the $LD_{50}$ for 791T cells there were the equivalent of $10^{11}$ VDS molecules and $1.5 \times 10^{10}$ antibody molecules per cell. It is possible that entry of the conjugate takes place during the 15 min pre-incubation period, but only at super-saturating levels.

In previous studies using a vindesine-polyclonal anti-CEA conjugate (Johnson *et al.*, 1982; Rowland *et al.*, 1981), it was found that a lung carcinoma cell line was much less susceptible to free vindesine than to conjugate. The present study shows no such difference; indeed free vindesine was somewhat more active. This contrast between the two studies cannot be readily explained, but may reflect differences in susceptibility of the cell lines used or may relate to the different properties of a monoclonal and a polyclonal antibody as a carrier.

Although it is difficult to predict the success of *in vivo* anti-tumour therapy on the basis of *in vitro* results, the above suggests that if VDS–α791T/36 could bind to tumour cells *in vivo*, then some anti-tumour response could be expected. It has been shown that radio-iodinated α791T/36 antibody localises specifically to xenografts of 791T, 788T 2 OS and T278 in immune-deprived mice (Pimm *et al.*, 1982*a*) so tumour localisation and binding of the drug-antibody conjugate would be likely to occur following parenteral injection. Since VDS is cytotoxic for cells in mitosis, where it disrupts formation of the mitotic spindle, it is likely to affect only cells regularly entering mitosis. In the case of xenografts it is possible that most cells fall into this category, but cells which are normally dormant and enter the mitotic subsequent to eradication of a portion of the tumour population could be subjected to a further treatment of the host with drug-antibody conjugate, if necessary.

Although repeated administration of murine monoclonal antibodies to patients might produce an anti-mouse Ig response, recent clinical studies (Sears *et al.*, 1982; Miller *et al.*, 1982) suggest that problems such as anaphylaxis can be avoided. If human monoclonals with suitable specificities become available (Baldwin *et al.*, 1981) some of the immune response problems should be eliminated. However, the drug portion of a conjugate may still act as an immunological hapten.

The main action of vindesine is as a phase-specific anti-mitotic agent. As such, when coupled to antibody, it offers a potential advantage over antibody-plant toxin conjugates which could kill any non-dividing cell binding the conjugate through antigenic cross-reactivity. Moreover, VDS is already in use clinically and its side-effects are considered to be tolerable.

It is not clear to what extent damage to the drug following chemical coupling would affect therapeutic efficacy of the conjugate. Although the drug could be expected to be less effective directly if administered at normal dosage, binding of the conjugate at the tumour site might counteract the loss of activity. In this case better conjugation methods, resulting in greater preservation of drug activity, could greatly enhance anti-tumour therapy. These questions can only be answered by *in vivo* experiments, and these are now being initiated using VDS–α791T/36 conjugate and xenografts arising from osteogenic sarcoma lines in immune-deprived mice.

M.J.E., E.J., and R.W.B., were supported by the Cancer Research Campaign, London, U.K.

We thank Mr C. Smith for help in purifying monoclonal antibody.

### References

BALDWIN, R.W., EMBLETON, M.J. & PRICE, M.R. (1981). Monoclonal antibodies specifying tumour-associated antigens and their potential for therapy. *Molec. Aspects Med*, **4**, 329.

BLYTHMAN, H.E., CASELLAS, P., GROS, O. & 5 others (1981). Immunotoxins: Hybrid molecules of monoclonal antibodies and a toxin subunit specifically kill tumour cells. *Nature*, **290**, 145.

EIKLID, K., OLSNES, S. & PIHL, A., (1980). Entry of lethal doses of abrin, ricin and modeccin into the cytosol of Hela cells. *Exp. Cell Res.*, **126**, 321.

EMBLETON, M.J., GUNN, B., BYERS, V.S., and BALDWIN, R.W. (1981). Antitumor reactions of monoclonal antibody against a human osteogenic sarcoma cell line, *Br. J. Cancer*, **43**, 582.

FAZEKAS DE ST. GROTH, S., (1979). 'The quality of antibodies and cellular receptors', In *Immunological Methods*, (Ed. Lefkovits & Perris) New York, Academic Press, p.l.

GILLILAND, D.G., STEPLEWSKI, Z., COLLIER, R.J., MITCHELL, K.F., CHANG, T.H., and KOPROWSKI, H. (1980). Antibody-directed cytotoxic agents: Use of monoclonal antibody to direct the action of toxin A-chains to colorectal carcinoma cells. *Proc. Nat. Acad. Sci.* **77**, 4539.

JOHNSON, J.R., FORD, C.H.J., NEWMAN, C.E., WOODHOUSE, C.S., ROWLAND, G.F., and SIMMONDS, R.G. (1982). A vindesine anti-CEA conjugate cytotoxic for human cancer cells *in vitro*. *Br. J.Cancer*, **44**, 472.

KROLICK, K.A., VILLIMEZ, C., ISAKSON, P., UHR, J.W., and VITETTA, E.S. (1980). Selective killing of normal or neoplastic B-cells by antibodies coupled to the A-chain of ricin. *Proc. Nat. Acad. Sci.*, **77**, 5419.

MILLER, R.A., MALONEY, D.G., WARNKE, R. and LEVY, R. (1982). Treatment of B-cell lymphoma with monoclonal anti-idiotype antibody. *N. Eng. J. of Med.* **306**, 517.

PIMM, M.V., EMBLETON, M.J., PERKINS, A.C. & 4 others (1982). *In vivo* localisation of anti-osteogenic sarcoma 791T monoclonal antibody in osteogenic sarcoma xenografts. *Int. J. Cancer*, **30**, 75.

PIMM, M.V., JONES, J.A., PRICE, M.R., MIDDLE, J.G., EMBLETON, M.J., and BALDWIN, R.B. (1982b). Tumour localisation of monoclonal antibody against a rat mammary carcinoma and suppression of tumour growth with Adriamycin-antibody conjugates. *Cancer Immunol. Immunother*, **12**, 125.

PRICE, M.R., PIMM, M.V., & BALDWIN, R.W. (1982). Complement-dependent cytotoxicity of anti-human osteogenic sarcoma monoclonal antibodies. *Br. J. Cancer*, **46**, 4.

ROWLAND, G.F., SIMMONDS, R.G., CORVALAN, J.R.F. & 5 others (1981). the potential use of monoclonal antibodies in drug targeting. *Protides Biol. Fluids*, **29**, 921.

SEARS, H.F., ATKINSON, B., MATTIS, J. & 5 others. (1982). Phase-1 clinical trial of monoclonal antibody in treatment of gastrointestinal tumours. *Lancet*, **i**, 762.

THORPE, P.E., & ROSS, W.C.J. (1982). The preparation and cytotoxic properties of antibody-toxin conjugates. *Immunol. Revs*, **62**, 119.

TROWBRIDGE, I.S., & DOMINGO, D.L. (1981). Anti-transferrin receptor monoclonal antibody and toxin-antibody conjugates affect growth of human tumour cells. *Nature*, **294**, 171.

YAMAIZUMI, M., MEKADA, E., UCHIDA, T. & OKADA, Y. (1978). One molecule of diphtheria toxin fragment A introduced into a cell can kill the cell. *Cell*, **15**, 245.